# Isolated posterior tibial artery thrombosis after non-severe SARS-CoV-2 infection

**DOI:** 10.1590/0037-8682-0205-2021

**Published:** 2021-06-02

**Authors:** Serdar Aslan, Tumay Bekci, Ismet Mirac Cakir

**Affiliations:** 1 Giresun University, Faculty of Medicine, Department of Radiology, Giresun, Turkey.

**FIGURE 1:**
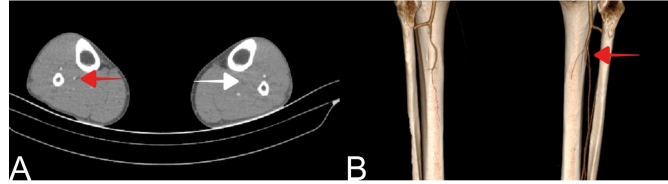
Left posterior tibial artery (PTA) thrombosis confirmed by computed tomography (CT) angiography and three-dimensional volume rendering images: (**A)** Shows the hypodense filling defect in the left PTA (white arrow), and the right PTA which appeared normal (red arrow); and (**B)** Similarly, three-dimensional volume-rendering CT images show that the right PTA is normal (red arrow) while the left PTA cannot be seen.

A 40-year-old man presented with left lower extremity pain for about 20 days, especially after walking; the pain was relieved by resting. He had a previous severe acute respiratory syndrome coronavirus 2 (SARS-CoV2) infection one month earlier, but it was not serious enough to require hospitalization. Apart from this, there was nothing significant in terms of atherosclerotic risk factors in his medical history. A Doppler ultrasound of the lower extremity showed no blood flow in the left posterior tibial artery (PTA). A thrombosis was confirmed in the left PTA by computed tomography angiography (CTA) and three-dimensional volume rendering images (Figure 1A and 1B). There was no thrombosis in or occlusion of the other vessels.

A predisposition to venous thrombosis has been reported in the literature, especially in severe cases of corona virus 2019. In reported cases of arterial thrombosis, the proximal vessels and large thrombus burden are frequently mentioned^1^. The present case is the first one in the literature to describe isolated PTA thrombosis associated with SARS-CoV2 infection. Therefore, we recommend considering the risk of vascular thrombosis in any case of SARS-CoV-2 infection. In cases of greater predisposition or severity, anticoagulant therapy should be considered^2^. To the best of our knowledge, anticoagulant treatment has few side effects, a reasonable cost burden, and may be instrumental in preventing the development of arterial thrombi and the related complications.
